# Mouse Hepatic Oval Cells Require Met-Dependent PI3K to Impair TGF-β-Induced Oxidative Stress and Apoptosis

**DOI:** 10.1371/journal.pone.0053108

**Published:** 2013-01-02

**Authors:** Adoración Martínez-Palacián, Gaelle del Castillo, Amileth Suárez-Causado, María García-Álvaro, Diego de la Morena-Frutos, Margarita Fernández, Cesáreo Roncero, Isabel Fabregat, Blanca Herrera, Aránzazu Sánchez

**Affiliations:** 1 Dep. Bioquímica y Biología Molecular II, Facultad de Farmacia, Universidad Complutense, Instituto de Investigación Sanitaria del Hospital Clínico San Carlos (IdISSC), Madrid, Spain; 2 Laboratori d´Oncologia Molecular and Departament de Ciències Fisiològiques II, Universitat de Barcelona, Institut d´Investigació Biomèdica de Bellvitge, ĹHospitalet de Llobregat, Barcelona, Spain; Osaka University Graduate School of Medicine, Japan

## Abstract

We have previously shown that oval cells harboring a genetically inactivated Met tyrosine kinase (Met^−/−^ oval cells) are more sensitive to TGF-β-induced apoptosis than cells expressing a functional Met (Met^flx/flx^), demonstrating that the HGF/Met axis plays a pivotal role in oval cell survival. Here, we have examined the mechanism behind this effect and have found that TGF-β induced a mitochondria-dependent apoptotic cell death in Met^flx/flx^ and Met^−/−^ oval cells, associated with a marked increase in levels of the BH3-only proteins Bim and Bmf. Bmf plays a key role during TGF-β-mediated apoptosis since knocking down of BMF significantly diminished the apoptotic response in Met^−/−^ oval cells. TGF-β also induced oxidative stress accompanied by NADPH oxidase 4 (Nox4) mRNA up-regulation and decreased protein levels of antioxidant enzymes. Antioxidants inhibit both TGF-β-induced caspase 3 activity and Bmf up-regulation, revealing an oxidative stress-dependent Bmf regulation by TGF-β. Notably, oxidative stress-related events were strongly amplified in Met^−/−^ oval cells, emphasizing the critical role of Met in promoting survival. Pharmacological inhibition of PI3K did impair HGF-driven protection from TGF-β-induced apoptosis and increased sensitivity of Met^flx/flx^ oval cells to TGF-ß by enhancing oxidative stress, reaching apoptotic indices similar to those obtained in Met^−/−^ oval cells. Interestingly, both PI3K inhibition and/or knockdown itself resulted in caspase-3 activation and loss of viability in Met^flx/flx^ oval cells, whereas no effect was observed in Met^−/−^ oval cells. Altogether, results presented here provide solid evidences that both paracrine and autocrine HGF/Met signaling requires PI3K to promote mouse hepatic oval cell survival against TGF-β-induced oxidative stress and apoptosis.

## Introduction

Oval cells constitute a bi-potential progenitor cell population from adult liver. When hepatocyte proliferation and/or function is impaired by chronic liver disease or hepatotoxin administration, oval cells emerge from the periportal area, in particular the canals of Hering, the terminal smallest branch of the biliary tree, and expand into the damaged parenchyma, a process known as “oval cell response” or “ductular reaction”. Oval cells can give rise to both hepatocytes and biliary epithelial cells and are characterized by the co-expression of both hepatocytic and cholangiocytic markers as well as hematopoietic and neuroepithelial markers, which reflects their immature phenotype [1], [2], [3]. These cells are nowadays a matter of intense investigation. On the one hand, due to their facultative role in liver regeneration, oval cells have been postulated as a therapeutic tool in acute or chronic liver diseases. On the other hand a link has been established between oval cells and hepatocarcinogenesis. Indeed, a substantial body of evidence in the literature supports the hypothesis that oval cells could be the origin of at least a subset of hepatocellular carcinoma (HCC) [4]. Understanding the intricate growth factor network and signaling events that regulate oval cell biology will help us to clarify these contrasting roles of oval cells in liver regeneration and tumor development.

Transforming growth factor beta (TGF-β), a member of the TGF-β superfamily ligands, has highly pleiotropic effects that depend on the dose, duration of signal and the type and state of the target cell. It has a crucial role during development, tissue remodelling and homeostasis by controlling many cellular processes, such as differentiation, proliferation, apoptosis and motility in many types of cells [5]. TGF-β initiates the intracellular signaling through binding to transmembrane serine-threonine kinase receptors and subsequent activation of SMAD proteins, which regulate gene expression [6]. Besides the canonical SMAD-mediated pathway, TGF-β triggers a variety of intracellular signaling pathways, commonly referred to as “non-SMAD” or “non-canonical” pathways that include mitogen-activated protein kinases (MAPKs), Rho-like guanosine triphosphatases (GTPases) and phosphatidilinositol-3-kinase PI3K/AKT pathways [7]. In hepatocytes, the best characterized effects of TGF-β are undoubtedly the induction of growth arrest and apoptosis, which target cells at different stages of differentiation and pathophysiological conditions, i.e. adult, fetal and regenerating hepatocytes [8], [9], [10], [11], [12]. Surprisingly, the effects of TGF-β on oval cell biology are not yet well defined. *In vivo* and *in vitro* data indicate that TGF-β negatively controls oval cell activation but the mechanisms underlying its effects have not been fully explored. Thus, transgenic mice expressing active TGF-β in the liver show an impaired oval cell response after hepatic chronic injury induced by a 3,5-diethoxycarbonyl-1,4-dihydro-collidine (DDC)-containing diet [13]. Furthermore, coinciding with the oval cell proliferation an increased expression of TGF-β1 in hepatic stellate cells is observed, followed by a peak in apoptosis of oval cells [14]. In agreement with these *in vivo* observations, TGF-β decreases rat oval cell growth *in vitro* although to a lesser extent than in hepatocytes [15]. We have also shown that TGF-β decreases cell viability and induces caspase-3 activation in oval cells *in vitro* [16]. Interestingly, CD133^+^/CD45^-^ oval cells isolated from methionine adenosyltransferase 1a (Mat1a^−/−^) deficient mice, a cell population that resemble cancer stem cells, show cell growth inhibition in response to TGF-β but appear to be resistant to its apoptotic effects [17].

Met is a proto-oncogene that encodes for the Hepatocyte Growth Factor (HGF) receptor. HGF binds to Met to mediate its multiple cellular activities including mitogenesis, motogenesis, morphogenesis and survival in a variety of cell types [18], [19]. Upon HGF binding, Met is activated and autophosphorylated in specific tyrosine residues, leading to the recruitment of signal transducers through the C-terminal docking site. Among the signaling pathways activated by Met in response to HGF are PI3K/AKT, phospholipase-C gamma (PLC-γ), sarcoma protein kinase (Src), signal transducer and activator of transcription 3 (STAT3), nuclear factor-kappa B (NF-κB) and MAPKs including Ras/extracellular signal-regulated kinases (ERKs), c-Jun N-terminal kinases (JNK) and p38 [19]. The HGF/Met signaling pathway is critical during tissue formation and homeostasis, playing a major role in liver physiology and pathology, both during development and adulthood. This notion is supported by the fact that deletion of either HGF or Met causes embryonic lethality due to multiple abnormalities including massive hepatoblasts apoptosis [20], [21]. Additionally, liver specific Met or HGF conditional knock-out mice show an impaired regenerative response associated with an increased cell death, delayed and decreased proliferation and healing [22], [23]. The increased apoptotic cell death is a common trait in all these genetic ablation models, highlighting the remarkable action of HGF/Met in the suppression of apoptosis. Antiapoptotic action of HGF/Met in liver does not exclusively target hepatocytes. To the contrary, HGF has proved to be a survival factor for oval cells against different apoptotic insults, such as Tumor Necrosis Factor (TNF-α), serum withdrawal and TGF-β [24], [25]. Accordingly, we have demonstrated that oval cells harboring a genetically inactivated Met tyrosine kinase (Met^−/−^ oval cells) are more sensitive to TGF-β-induced apoptosis than their normal counterparts (Met^flx/flx^ oval cells) [24].

Seeking to understand how HGF/Met exerts its survival effect against TGF-β-induced apoptosis, we conducted a broad analysis on the apoptotic signaling cascade induced by TGF-β in both Met^flx/flx^ and Met^−/−^ oval cell lines. Here we report that TGF-β induces a mitochondrial apoptotic cell death in oval cells, with up-regulation of BIM and BMF proteins, two BH3-only members of the Bcl-2 family. Furthermore, we reveal a signaling pathway in which TGF-β induces oxidative stress associated with up-regulation of *Nox4* and down-regulation of the intracellular antioxidant defenses, which leads to *Bmf* up-regulation and subsequent cell apoptosis. Although both Met^flx/flx^ and Met^−/−^ oval cells do respond to TGF-β, alteration of both mitochondrial function and oxidative homeostasis are amplified in Met^−/−^ oval cells, providing one mechanism for the increased sensitivity to TGF-β-triggered apoptosis in Met-deficient oval cells. Finally, our results provide strong evidence that PI3K may be a key player in mediating anti-apoptotic signals via Met in oval cells by acting as an antioxidant signal.

## Materials and Methods

### Reagents and Antibodies

Mouse recombinant HGF was purchased from R&D Systems (Minneapolis, MN). Human recombinant TGF-β, ERK inhibitor PD90059, p38 inhibitor SB203580 and PI3K inhibitor LY294002 were from Calbiochem (La Jolla, CA). SP600125 JNK inhibitor was from Alexis Biochemical (Madrid, Spain), Dulbecco’s modified Eagle’s medium (DMEM), fetal bovine serum (FBS) and trypsin-EDTA were from Gibco-Invitrogen (Barcelona, Spain). Ascorbate, pyrrolidine carbodithioic acid (PDTC), penicillin, streptomycin, HEPES, bovine serum albumin (fraction V, fatty-acid free), propidium iodide, DNA oligos and buffer reagents were from Sigma-Aldrich (Tres Cantos, Madrid, Spain). 2′,7′-dichlorofluorescein-diacetate (DCFH-DA) was from Molecular Probes (Eugene, OR). RNeasy Kit was from Qiagen (Valencia, CA). SuperScript III RNase H Reverse Transcriptase was from Invitrogen. Oligo-dT was from Roche Diagnostics (Sant Cugat del Valles, Barcelona, Spain). Horseradish peroxidase-conjugated secondary antibody and ECL reagent were from GE Healthcare Europe (Barcelona, Spain). Caspase-3 substrate was obtained from PharMingen (San Diego, CA).

The rabbit polyclonal antibodies against phospho-Smad2 (Ser 465/467) (CS3101), phospho-p38 (Thr180/Tyr1829) (CS9211) and GADPH (CS2118) were purchased from Cell Signaling (Beverly, MA). Rabbit polyclonal against p38 (SC-535) and mouse monoclonal against phospho-JNK (SC-6254) antibodies were from Santa Cruz Biotechnology, Inc., (Paso Robles, CA). Mouse monoclonal anti-Cytochrome C (556433) and rabbit polyclonal anti-Bim (559685) and anti-Bcl-x (610211) antibodies were from BD Biosciences. Anti-β-actin (clone AC-15) and anti-Catalase (C0979) mouse monoclonal antibodies were from Sigma-Aldrich. Polyclonal antibodies anti-Manganese Superoxide Dismutase 2 (SOD2) (06–984) and anti-PI3K p85 (06–195) were from Millipore, anti-gamma-Glutamylcysteine Synthetase (γ-GCS) from Abcam (40929) and mouse monoclonal antibody anti-Bmf from Alexis Biochemicals (ALX-804-342).

### Cell Lines and Culture Conditions

Met^flx/flx^ and Met^−/−^ oval cell lines were generated as described previously [24]. Cells were routinely maintained in DMEM supplemented with 10% FBS in a humidified incubator at 37°C and a 5% CO_2_ atmosphere. Medium was replaced every three days, and cells were harvested at 80% to 90% confluence using trypsin-EDTA and replated at 1∶10 dilution for maintenance. After an overnight attachment period, medium was replaced by serum-free DMEM. Cells were maintained in serum-free medium for 4–12 hours prior to treatment with growth factors. Where indicated, cells were pretreated with HGF for at least 6 hours followed by TGF-β treatment. PD98059, SB203580, LY294002, SP600125, ascorbate and PDTC were added 30 minutes before addition of growth factors.

### Analysis of Apoptosis by Phosphatidylserine Exposure

Cells were collected by centrifugation at 1300 rpm for 5 min and washed once with PBS. 500,000 cells were resuspended with 195 µl of binding buffer (10 mM HEPES, pH 7.4, 2.5 mM CaCl_2_, 140 mM NaCl) supplemented with 5 µl annexin V-FITC (BD Pharmingen) and incubated for 10 min at room temperature. Samples were centrifuged and resuspended with 300 µl of binding buffer containing 1 µg/ml propidium iodide. Fluorescence intensity was analyzed using a FACSCalibur flow cytometer. 10,000 cells were recorded in each analysis.

### Measurement of Intracellular ROS

For the analysis of intracellular ROS by flow cytometry, the oxidation-sensitive probe DCFH-DA was used, as previously described [12]. Cells were detached by trypsinization, collected by centrifugation at 1300 rpm for 5 min and washed once with PBS. Samples were then incubated with 5 µM DCFH-DA for 30 minutes at 37°C. Cellular fluorescence intensity was measured in a FACScan flow cytometer. Propidium iodide (0.005%) was used to detect dead cells. For each analysis 10,000 events were recorded.

### Glutathione Determination

Cells were washed twice with PBS, scraped off and pelleted at 4°C. Cellular glutathione was extracted in a buffer containing 0.2% Triton X-100, 2.5% sulfosalicylic acid. After centrifugation at 12000 rpm for 15 min at 4°C, the supernatant was used for the determination of total glutathione, using the method of Griffith modified as described previously [26]. Using reduced glutathione as standard, glutathione content is expressed as nmol/µg protein.

### Analysis of Mitochondrial Transmembrane Potential

Cells were collected by centrifugation at 1300 rpm for 5 min, washed once and incubated with 2 µM 5,5′,6,6′-tetrachloro-1,1′,3,3′-tetraethylbenzimidazolylcarbocyanine iodide (JC-1) (Molecular Probes) for 1 hour at 37°C. Then, samples were centrifuged and resuspended with 500 µl DMEM. Fluorescence intensity (FL1 and FL2) was measured in a FACScan flow cytometer. 10,000 cells were recorded in each analysis. JC-1 dye exhibits potential-dependent accumulation in mitochondria. Cells with healthy mitochondria show both red (FL2) (aggregate, mitochondrial) and green (monomeric, cytoplasmic) fluorescence. Mitochondria depolarization is indicated by a decrease in the red/green (FL2/FL1) fluorescence intensity ratio, which is due to loss of red J-aggregate fluorescence and cytoplasmic diffusion of green monomer fluorescence.

### Transcriptional Reporter Assays

Transcriptional reporter assays were performed using Cignal SMAD Reporter (luc) kit (CCS-017L) from Qiagen. Cells were plated in 96 well dishes. The next day, DNA was transfected into the cells with Fugene transfection reagent, according to manufacturer recommendations. After 15 hours, medium was replaced with serum-free medium for additional 15 hours. Cells were then incubated with or without TGF-β and firefly and renilla (normalizing transfection control) luciferase activities were measured using “Dual luciferase Reporter Assay System” (Promega) in a luminometer Fluostar Omega (BMG labtech).

### Quantitative Reverse Transcriptase-Polymerase Chain Reaction (qRT-PCR) Analysis

Total cellular RNA was isolated using the RNeasy Kit (Qiagen, Valencia, CA). RNA yield and purity were analyzed using a spectrophotometer (UV-visible recording spectrophotometer Specord 205, AnalytikJena). 1 µg total RNA was reverse-transcribed into complementary DNA using SuperScript III RNase H Reverse Transcriptase and oligo-dT as a primer. Quantitative PCR was performed using SYBRGREEN (Roche) and Amplified products were analysed in ABI Prism 7900 HT Fast Real-Time (Applied Biosystems). The relative amount of target mRNA was determined after normalization against reference gene (*Gusb*) in each sample. Primers used in the study are presented in supporting information, table S1.

### Measurement of Caspase-3-Like Enzymatic Activity

A fluorometric assay in the presence of Ac-DEVD-AMC as fluorogenic Caspase-3 substrate was used following a previously described procedure [26]. Cleavage of the substrate was monitored in a Microplate Fluorescence Reader FL600 (Bio-Tek) (excitation, 380 nm; emission, 440 nm). A unit of caspase activity is the amount of enzyme that will lead to a one unit increase in the fluorescence intensity. Protein concentration was estimated and results are expressed as units of activity per microgram of protein.

### Immunoblotting

For standard western blotting total cell extracts were prepared in modified RIPA buffer (30 mM Tris pH 7.5; 150 mM sodium chloride; 1% NP40; 0.5% sodium deoxycholate; 0.1% SDS; 5 mM EDTA) supplemented with 1 mM phenylmethylsulfonyl fluoride, 10 µg/ml aprotinin and leupeptin and 1 mM sodium orthovanadate. 30 to 80 µg of protein were separated in 10–12% acrylamide sodium dodecyl sulfate-polyacrylamide electrophoresis gels and blotted to Immobilon-P membranes (Millipore, Bedford, MA). Membranes were probed with the primary antibodies diluted 1∶500 to 1∶1000 in Tris-buffered saline containing 0.1% Tween 20 and 0.5% non-fat dried milk or 0.5% bovine serum albumin according to manufacturer’s instructions. Detection was done using the enhanced chemiluminescence method and autoradiography.

### Measurement of Apoptotic Index

Measurement of apoptotic index was performed as previously described [24]. After staining with propidium iodide, cells undergoing apoptosis were scored under inverted fluorescence microscope (Eclipse TE300, Nikon) at high magnification (x60) following standard morphological criteria. Apoptotic indices were calculated after counting a minimum of 1000 cells per treatment in a blinded manner.

### siRNA Knockdown Assays

siRNA knockdown assays were performed as previously described [27]. For transient siRNA transfection, cells were seeded at 50–60% confluence. On the following day, cells were transfected with a SMARTpool siRNA directed to the mouse p85α regulatory subunit of PI3K (50 nM) or to mouse Bmf (20 nM) or the control non-targeting siRNA (50 nM and 20 nM, respectively) (Dharmacon, Lafayette, CO) using TransIT-siQuest reagent (Mirus, Madison, WI) according to manufactureŕs instructions. Transfected cells were grown for 24 hours in complete medium, then tripsinized, diluted to the appropriate cell density, and replated in dishes for subsequent assays.

### Adenovirus-mediated Met Knockdown

*In vitro* inactivation of Met was achieved following previous protocols [24]. Twenty-four hours after plating, Met^flx/flx^ cells were infected with adenovirus expressing Cre recombinase (Ad-CMVCre, Vector Biolabs, Philadelphia, PA) to disrupt endogenous *met* allele, or an empty adenovirus (Ad-CMVNull) used as control. Virus was diluted in infection medium (growth medium supplemented with 2.5 µg/ml polybrene) at a multiplicity of infection of 10. Cells were incubated in infection medium for one hour in the incubator with occasional shaking and then fresh medium was added to complete volume. After 24 hours of infection, cells were serum starved for 4 hours and treated with TGF-β for additional 24 hours to analyze cell apoptosis. In parallel, infected cells were used to isolate genomic DNA following standard procedures to identify the deleted allele by PCR using specific oligonucleotides [23].

### Statistical Analysis

Statistical analysis was performed by Studentś*t*-test method. The differences were assumed significant at *P*<0.05.

## Results

### Absence of a Functional Met Receptor does not Affect TGF-β Induced Activation of Canonical and Non-canonical Signaling Pathways in Oval Cells

**Figure 1:**
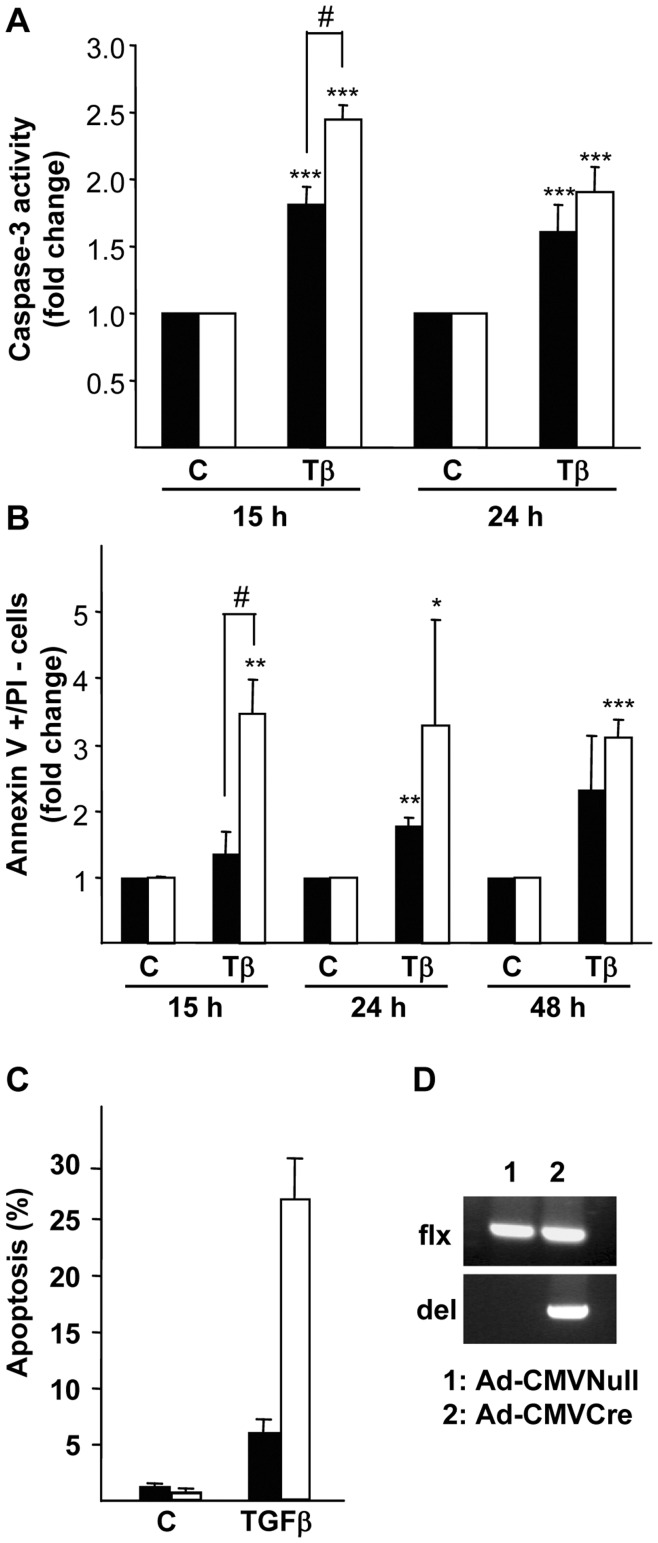
Increased sensitivity to TGF-β-induced apoptosis in oval cells lacking a functional Met receptor. A. Mouse Met^flx/flx^ and Met^−/−^ oval cell lines were serum-starved and incubated in the absence (C) or presence of 1 ng/ml TGF-β (Tβ) for different periods of time. Cells were lysed and caspase-3 activity was measured. Data are mean±SEM of eight independent experiments. **B.** Cells were treated as in A, detached by tripsinization and incubated with annexin V and PI. Subsequently, fluorescence intensity was measured in a FACScan flow cytometer and the percentage of annexin V positive/PI negative cells was calculated. Data are expressed as fold induction over untreated cells and are mean±SEM of four independent experiments. **C.** Cells were infected with Ad-CMVCre or Ad-CMVNull for 24 hours, then serum-starved for 4 hours and treated with 1 ng/ml TGF-β for 24 hours. Apoptotic nuclei were visualized and counted after PI staining under a fluorescence microscope. A minimum of 1000 nuclei was counted per condition. **D.** PCR genotyping of genomic DNA to confirm *met* deletion in infected cells. **Black bars**, Met^flx/flx^ cells. **White bars**, Met^−/−^ cells. *P<0.05; **P<0.01; ***P<0.001 (treated versus untreated); #*P*<0.05 (treated Met^−/−^ versus treated Met^flx/flx^).

**Figure 2:**
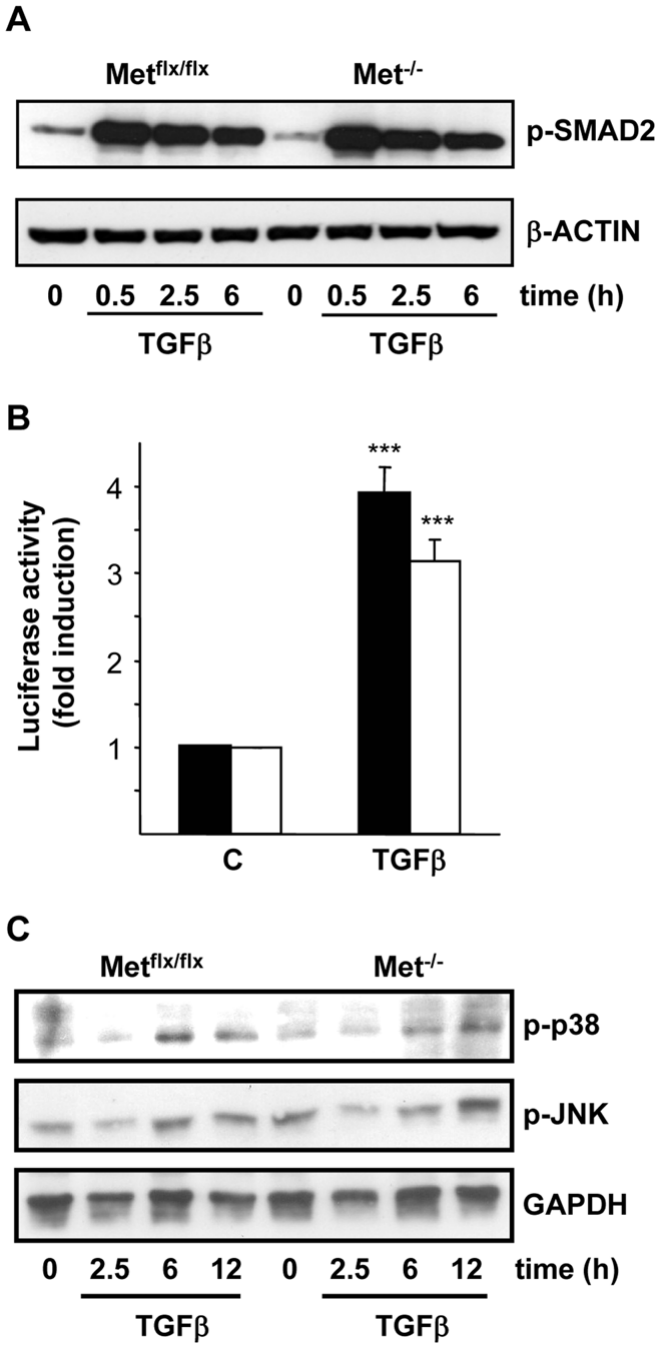
Comparison of the TGF-β-induced canonical and non-canonical signaling in oval cell expressing a functionally active or inactive Met receptor. A. Mouse Met^flx/flx^ and Met^−/−^ oval cell lines were serum-starved and treated with 1 ng/ml TGF-β for different periods of time as indicated. Untreated cells were included as control. Whole cell lysates were collected and used for immunoblotting with anti-phosphoSmad2 antibody. β-actin was analyzed as loading control. A representative experiment of two is shown. **B.** Mouse Met^flx/flx^ and Met^−/−^ oval cell lines were transiently transfected with SBE containing construct linked to a luciferase reporter (Cignal Smad reporter). Cells were serum-starved and incubated in the absence (C) or presence of 1 ng/ml TGF-β (Tβ) for 8 hours. Normalized luciferase activity is shown as fold induction relative to untreated cells. Data are mean±SEM of three independent experiments run in triplicates. **C.** Cells were treated as in A. Whole cell lysates were collected and used for immunoblotting with indicated antibodies. A representative experiment of two is shown. **Black bars**, Met^flx/flx^ cells. **White bars**, Met^−/−^ cells. ***P<0.001 (treated versus untreated).

Previous results from our group have shown that mouse oval cell lines harboring a genetically inactivated Met tyrosine kinase (Met^−/−^) respond differently to apoptotic insults than Met^flx/flx^ oval cells and show an increased sensitivity to TGF-β and serum starvation-induced apoptosis [24]. Indeed, caspase-3 activity increased in oval cells upon TGF-β addition, being this increase significantly higher in Met^−/−^ than in Met^flx/flx^ oval cells (figure 1A). Caspase-3 activation occurred concomitantly with other apoptotic hallmarks. Thus, TGF-β promoted phosphatilserine exposure on the outer leaflet of the plasma membrane, which started after 15 hours of treatment and was maintained for several hours (figure 1B). It is noteworthy that the annexin V positive/propidium iodide negative subpopulation, corresponding to the apoptotic cell subpopulation, was larger in Met^−/−^ oval cells compared to their normal counterparts, serving as additional proof that absence of a functional Met sensitizes oval cells to apoptosis. As an alternative approach Met was transiently knocked down in oval cells by infection with adenovirus expressing Cre recombinase under a CMV promoter (Ad-CMVCre). Subsequently, cells were treated with TGF-β and apoptosis was measured. Cells infected with the Ad-CMVCre virus elicited a stronger apoptotic response to TGF-β compared to control cells infected with an empty adenovirus vector (Ad-CMVNull) (figure 1C) demonstrating the specificity of the changes observed in the cell lines. Aiming to understand the functional relevance of Met in the TGF-β-triggered apoptosis in oval cells, we next study whether Met mutant cells have an altered signaling response to TGF-β. In rat oval cells, TGF-β induces phosphorylation and nuclear translocation of SMAD2 [15] but nothing is known about activation of other signaling pathways by this factor in oval cells. On this basis, we first checked TGF-β ability to induce SMAD2 phosphorylation in mouse oval cells by western blot. SMAD2 phosphorylation was maximal after 30 minutes of treatment with TGF-β and was maintained up to 6 hours. No differences in kinetics or intensity of SMAD2 phosphorylation were detected between Met^flx/flx^ and Met^−/−^ oval cells (figure 2A). Consistently, nuclear p-SMAD2 protein levels were similarly increased upon TGF-β treatment in Met^flx/flx^ and Met^−/−^ oval cells (data not shown). To further demonstrate SMAD-dependent signaling in both cell lines, transcriptional reporter assays were performed using a commercial construct containing Smad Binding Elements (SBE) linked to luciferase, which can be considered as a read out for transcriptional responses to TGF-β. Cells were transfected with SBE-containing construct and incubated in the presence or absence of TGF-β for 8 hours. A three to four-fold induction in luciferase reporter activity was observed upon TGF-β treatment in both Met^flx/flx^ and Met^−/−^ oval cells (figure 2B). Altogether, these results indicate that activation of the SMAD pathway in response to TGF-β in mouse oval cells is not affected by the absence of a functional Met. TGF-β also activates other non-SMAD signaling pathways [7]. Among them, p38 and JNK MAPKs pathways have been reported to play a relevant role in controlling the apoptotic response to TGF-β in a variety of cell types [28]. Therefore, we next checked whether TGF-β triggers these signaling cascades by western blot analysis using antibodies against the phosphorylated (active) form of p38 and JNK. Results in figure 2C show that treatment with TGF-β activates JNK and p38 in both Met^flx/flx^ and Met^−/−^ cells and no major differences were observed between cell lines. To evaluate whether these kinases are involved in TGF-β-mediated apoptotic response in mouse oval cells, Met^flx/flx^ and Met^−/−^ cells were preincubated with the synthetic p38 inhibitor SB203580 or JNK inhibitor SP600125 and subsequently treated with TGF-β. TGF-β was able to increase caspase-3 activity regardless of presence or absence of the inhibitors (supporting information, figure S1), demonstrating that neither p38 nor JNK activation is required for the TGF-β apoptotic effect in oval cells. Whether these signal effectors are involved in other biological responses to TGF-β in oval cells remains to be investigated.

### Mitochondrial Apoptotic Program Triggered by TGF-β is Enhanced in Oval Cells Lacking a Functional Met

**Figure 3:**
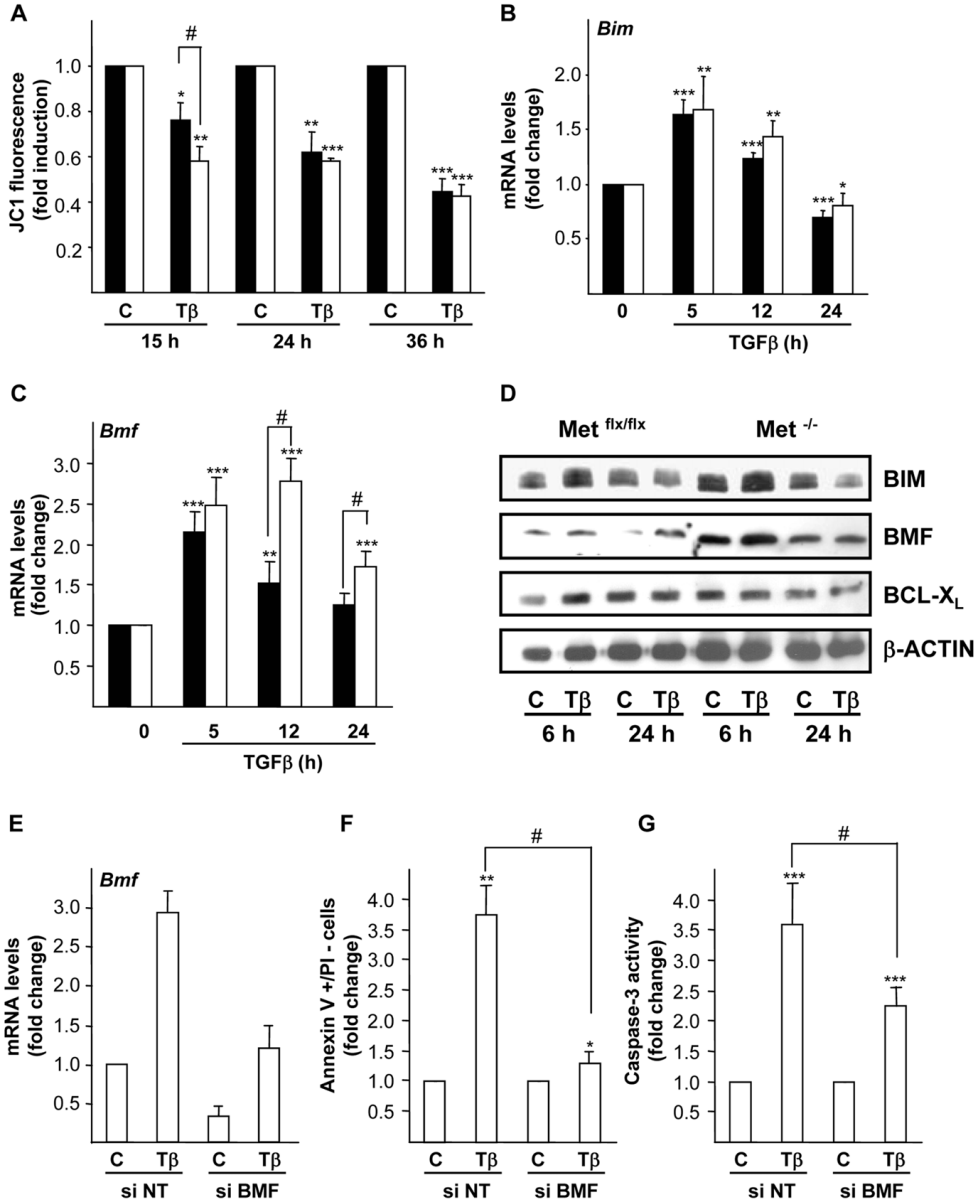
TGF-β induces mitochondrial depolarization and changes in expression of Bcl-2 family members in oval cells. Mouse Met^flx/flx^ and Met^−/−^ oval cell lines were serum-starved and incubated in the absence (C) or presence of 1 ng/ml TGF-β (Tβ) for different periods of time. **A.** After 1 hour incubation with JC1 (2 µM), fluorescence intensity (FL1 and FL2) was measured in a FACScan flow cytometer. Results are expressed as FL2/FL1 fluorescence intensity ratio, as indicative of changes in the mitochondrial membrane potential. Data are mean±SEM of three independent experiments run in duplicate. *P<0.05; **P<0.01; ***P<0.001 (treated versus untreated), #*P*<0.05 (treated Met^−/−^ versus treated Met^flx/flx^). **B and C.** *Bim* and *Bmf* mRNA levels were analyzed by qRT-PCR and normalized to the housekeeping gene *Gusb*. Data represent fold change relative to untreated samples and are mean±S.E.M of at least four independent experiments. *P<0.05; **P<0.01; ***P<0.001 (treated versus untreated), #*P*<0.05 (treated Met^−/−^ versus treated Met^flx/flx^). **D**. Whole cell lysates were collected and used for immunoblotting with the indicated antibodies. β-actin was analyzed as loading control. A representative experiment is shown. **E, F and G.** Mouse Met^−/−^ oval cells were transfected with either non-targeting negative control siRNA (si NT) or BMF targeting siRNA (si BMF). **E.** Twenty-four hours after transfection, cells were serum starved and treated or not with TGF-β (1 ng/ml) for 5 hours. RNA was isolated and *Bmf* mRNA levels were analyzed by qRT-PCR and normalized to the housekeeping gene *Gusb*. Data represent fold change relative to si NT untreated cells and are mean±S.D of a representative experiment out of two. **F.** Twenty-four hours after transfection, cells were serum starved and treated or not with TGF-β (1 ng/ml) for 24 hours. Fluorescence intensity was measured in a FACScan flow cytometer and the percentage of annexin V positive/PI negative cells was calculated. Data are expressed as fold induction over untreated cells and are mean±SD of a representative experiment out of two. **G**. Twenty-four hours after transfection, cells were serum starved and treated or not with TGF-β (1 ng/ml) for 15 hours. Cells were lysed and caspase-3 activity was measured. Data are mean±SEM of three independent experiments. *P<0.05; **P<0.01; ***P<0.001 (treated versus untreated), #*P*<0.05 (as indicated). **Black bars**, Met^flx/flx^ cells. **White bars**, Met^−/−^ cells.

Our next goal was to perform a detailed analysis of the apoptotic cascade induced by TGF-β in oval cells. TGF-β has been described to induce mitochondrial apoptosis in different cells types including hepatocytes and HCC cells [26], [28], [29]. To assess if mitochondria was implicated in the apoptosis induced by TGF-β in mouse oval cells, we analyzed changes in the mitochondrial inner transmembrane potential (ΔΨ_m_) by flow cytometry after incubation of the cells with the fluorescent probe JC1. TGF-β provoked a decrease in ΔΨ_m_ both in Met^flx/flx^ and Met^−/−^ cells (figure 3A) that was accompanied by cytochrome c release from the mitochondria to the cytosol (data not shown). Importantly, our data revealed that this effect was magnified in Met^−/−^ cells since the dissipation of ΔΨ_m_ was significantly lower in Met^−/−^ oval cells than in Met^flx/flx^ cells at the earlier time point analyzed (15 hours of treatment), which is consistent with an increased caspase-3 activity in the Met^−/−^ oval cells (figure 1A). Since Bcl-2 family members act as sentinels of mitochondrial outer membrane permeabilization (MOMP) in the mitochondrial apoptotic pathway [30], we next tested the possibility that TGF-β could modulate the expression of some members of this family. In fact, it has been previously described that TGF-β up-regulates the pro-apoptotic Bcl-2 family members *Bim* and *Bmf* [31], [32], [33] and down-regulates the anti-apoptotic member *Bcl-x_L_* [26] at the transcriptional level. In oval cells both *Bim* and *Bmf* mRNA levels were up-regulated by TGF-β (figure 3B and 3C). *Bim* mRNA levels increased rapidly after treatment reaching maximum levels after 5 hours and dropped to basal levels or even below after 12 and 24 hours, respectively, in both cell lines. *Bmf* mRNA expression follows similar kinetics in Met^flx/flx^ cells, but interestingly, up-regulation of *Bmf* mRNA was significantly stronger in Met^−/−^ cells and remained elevated longer than in Met^flx/flx^ cells. Data were confirmed at the protein level, which showed an early increase in BIM upon TGF-β treatment (6 hours) both in Met^flx/flx^ and Met^−/−^ cells that display similar maximum levels. BMF was also up-regulated in both cell types, but showed much higher levels in Met^−/−^ oval cells. It is worth noting that basal levels of BIM and BMF were higher in Met^−/−^ cells than in Met^flx/flx^ cells, which is likely related to the increased apoptosis observed in oval cells lacking a functional Met under serum deprivation [34]. No significant modulation of BCL-X_L_ (figure 3D) was observed upon TGF-β treatment. Altogether, these results evidence a role for the mitochondria in the apoptotic pathway induced by TGF-β in oval cells. Remarkably, absence of a functional Met in oval cells results in an enhancement of the mitochondrial apoptotic events, such as dissipation of ΔΨ_m_ and Bmf up-regulation induced by TGF-β. In fact, differences in Bmf regulation suggested an important role for this protein in TGF-β-mediated apoptosis in oval cells, and therefore in amplification of the apoptotic response observed in Met^−/−^ oval cells. To evaluate this hypothesis, we performed transient knockdown experiments using specific siRNA targeting Bmf in Met^−/−^ oval cells. A Bmf silencing efficiency of 70% (figure 3E) significantly reduced TGF-β-induced apoptosis in oval cells, as demonstrated by a clear decrease in the percentage of apoptotic cells (annexin-V positive/IP negative cells) measured by flow cytometry (figure 3F) and a significant reduction in caspase-3 activity (figure 3G). These results served as unequivocal proof that Bmf plays an essential role in the TGF-β-triggered apoptotic signaling cascade in oval cells.

### TGF-β Induces Oxidative Stress in Oval Cells that is Amplified in the Absence of a Functional Met

**Figure 4:**
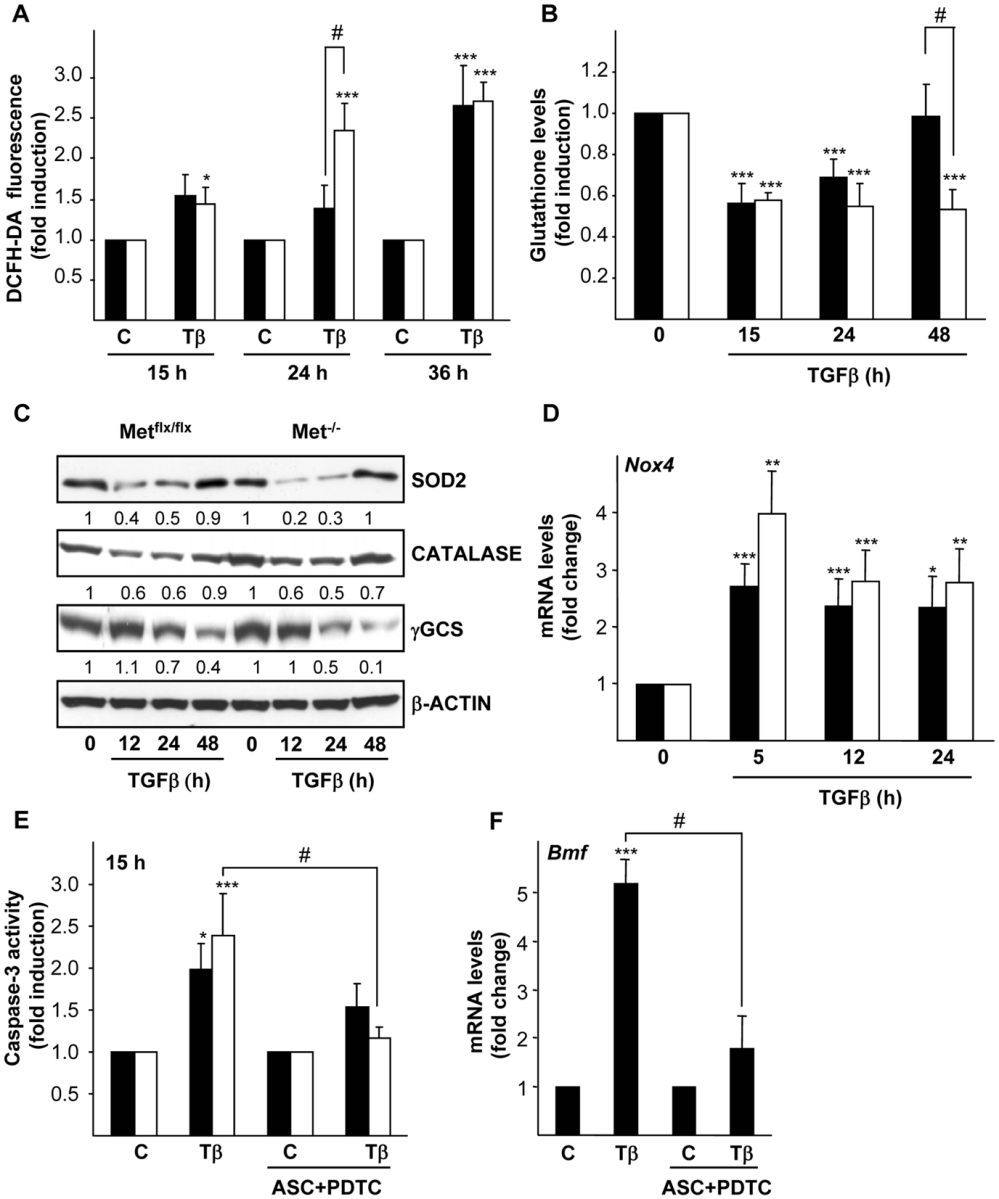
Intracellular oxidative stress induced by TGF-β in oval cells is amplified in cells lacking a functional Met receptor. Mouse Met^flx/flx^ and Met^−/−^ oval cell lines were serum-starved and incubated in the absence (C) or presence of 1 ng/ml TGF-β (Tβ) for different periods of time. **A.** After 30 minutes incubation with DFCH-DA (5 µM) fluorescence intensity was measured in a FACScan flow cytometer. Data are expressed as fold induction over untreated cells and are mean±SEM of three independent experiments run in duplicate. **B.** Cells were collected for spectrophotometric determination of intracellular glutathione. Results are expressed as fold change over untreated cells and are mean±SEM of three independent experiments run in duplicate. *P<0.05; **P<0.01; ***P<0.001 (treated versus untreated); #*P*<0.05 (treated Met^−/−^ versus treated Met^flx/flx^). **C.** Whole cell lysates were collected and used for immunoblotting with indicated antibodies. β-actin was analyzed as loading control. Optical density values relative to loading control were calculated. A representative experiment of two is shown. **D.** RNA was isolated and *Nox4* mRNA levels were analyzed by qRT-PCR and normalized to the housekeeping gene *Gusb*. Data represent fold change relative to untreated samples and are mean±S.E.M of at least four independent experiments. *P<0.05; **P<0.01; ***P<0.001 (treated versus untreated). **E.** Cells were pretreated or not with radical scavengers (1 mM ascorbate +50 µM PDTC) for 1 hour followed by treatment with TGF-β for 15 hours. Cells were lysed and caspase-3 activity was measured. Data are expressed as fold induction relative to untreated cells and are mean±SEM of three independent experiments run in duplicate. **F.** Mouse Met^flx/flx^ oval cells were pretreated or not with radical scavengers (1 mM ascorbate +50 µM PDTC) for 1 hour prior to TGF-β treatment (1 ng/ml). After 5 hours, RNA was isolated and *Bmf* mRNA levels were analyzed by qRT-PCR and normalized to the housekeeping gene *Gusb*. Data represent fold change relative to untreated samples and are mean±S.D from one representative experiment out of two. *P<0.05; **P<0.01; ***P<0.001 (treated versus untreated); #*P*<0.05 (TGF-β treated versus ASC+PDTC+TGF-β treated). **Black bars**, Met^flx/flx^ cells. **White bars**, Met^−/−^ cells.

ROS production is an early event of the apoptotic process induced by TGF-β in a number of epithelial cells, including normal and transformed hepatocytes [12], [26], [31], [35], [36]. We hypothesized that TGF-β might act through a similar mechanism in oval cells. Indeed, results shown in figure 4A indicate that TGF-β increases intracellular ROS content as measured by flow cytometry after incubation with the fluorescent probe DCFH-DA. ROS increase was observed in both cell lines but was significantly larger in Met^−/−^ cells after 24 hours of TGF-β treatment. Intracellular ROS accumulation correlated with a depletion of glutathione levels that occurred in both cell lines upon TGF-β treatment (figure 4B). It is noteworthy that while in Met^flx/flx^ cells glutathione content completely recovered to the normal levels after 48 hours, in Met^−/−^ cells glutathione levels still remained low. To further characterize the oxidative stress process induced by TGF-β in these cells, we analyzed the expression of antioxidant enzymes such as mitochondrial MnSOD (SOD2), catalase and γ-GCS. SOD activity is the major intracellular antioxidant defense against superoxide anion, being SOD2 one of the three mammalian SOD isoforms. Catalase catalyzed the decomposition of hydrogen peroxide to water and oxygen while γ-GCS is the rate limiting enzyme of the glutathione biosynthesis pathway [37]. SOD2, catalase and γ-GCS protein levels were strongly down-regulated upon TGF-β challenge (figure 4C). SOD2 down-regulation by TGF-β was also observed at the mRNA levels (data not shown), indicating a TGF-β-mediated transcriptional regulation of *Sod2* gene as previously described [38]. Importantly, coincident with accentuated ROS increase and glutathione depletion, Met^−/−^ oval cells display a stronger reduction in SOD2 and γ-GCS protein levels after TGF-β treatment as compared to their normal counterparts, suggesting that Met helps counteract loss of antioxidant defenses induced by TGF-β. Besides antioxidant enzymes down-regulation, previous data of our group had reported the implication of the NADPH oxidase Nox4 in TGF-β-triggered ROS production and apoptosis in hepatocytes [31], [36], [39]. We analyzed *Nox4* expression in oval cells and found an early and sustained up-regulation of *Nox4* mRNA by TGF-β (figure 4D) that precedes ROS accumulation (figure 4A) suggesting a role for Nox4 as a source of ROS in oval cells. Met^−/−^ oval cells display higher up-regulation of *Nox4* mRNA than Met^flx/flx^ cells, although differences did not reach statistical significance. Next, we raised the question of whether ROS production induced by TGF-β was involved in the apoptotic response of oval cells. To answer this question, cells were incubated with ascorbate and PDTC, two radical scavenger agents that have previously proved to be very efficient in counteracting ROS effects in fetal hepatocytes [12], [26]. Indeed, pretreatment of oval cells with these antioxidant agents completely abrogated ROS accumulation induced by TGF-β (supporting information, figure S2), and more importantly, significantly inhibited TGF-β-induced caspase-3 activity (figure 4E) demonstrating their capacity to counteract the apoptotic response. Additionally, we analyzed the effect of antioxidants on *Bmf* and *Bim* mRNA expression levels (figure 4F and data not shown, respectively) and found that antioxidants were able to inhibit TGF-β-mediated induction of *Bmf*, but not *Bim*. Collectively, these results show that TGF-β induces apoptosis in oval cells through an oxidative stress process involving Bmf induction. Furthermore, we provide strong evidence of a Met-mediated antioxidant protective effect in oval cells.

### PI3K is Required for HGF Anti-apoptotic Activity in Oval Cells

**Figure 5:**
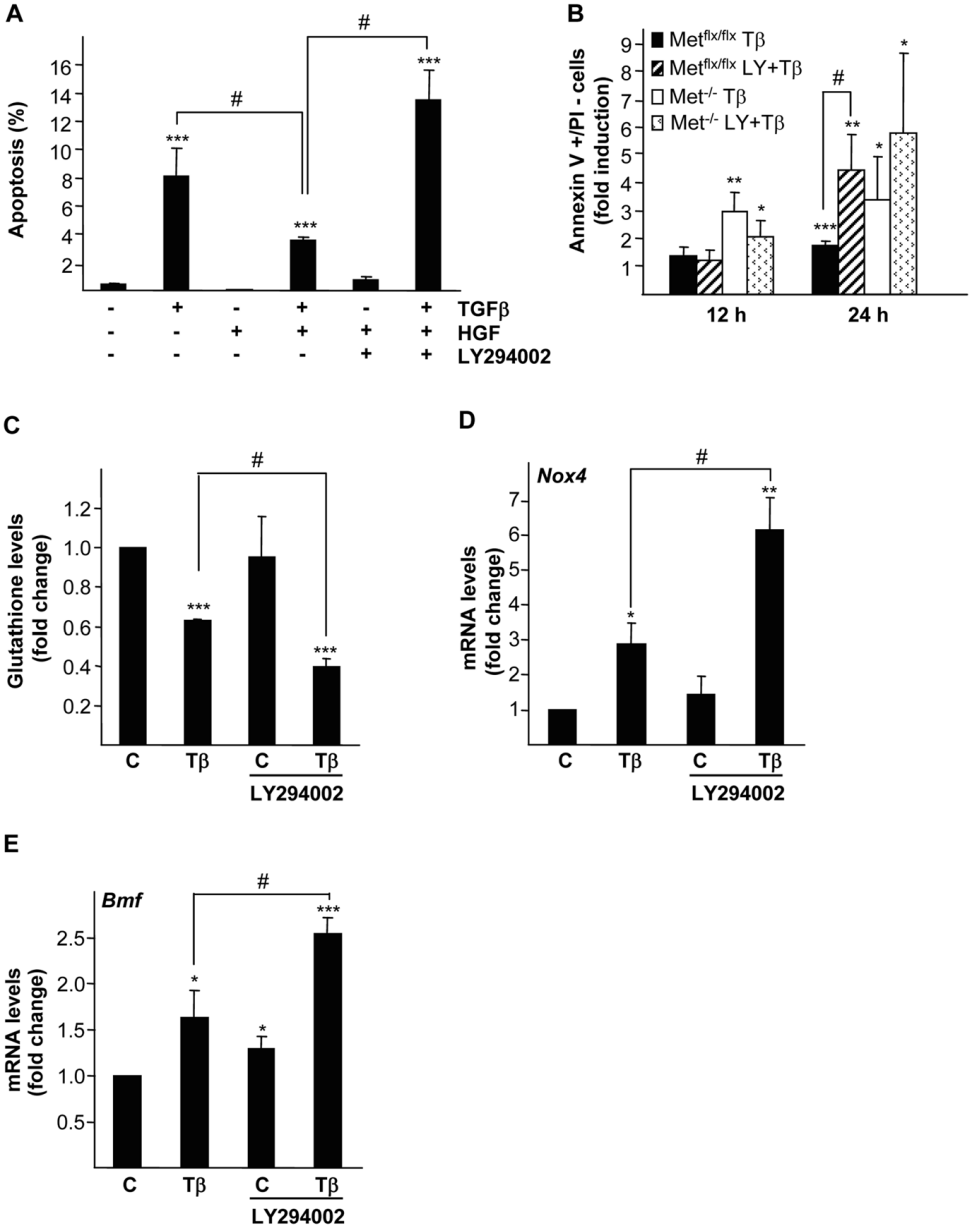
Effect of PI3K inhibition on TGF-β-induced apoptotic and HGF-induced anti-apoptotic activities in oval cells. A. Mouse Met^flx/flx^ oval cells were serum-starved and incubated in the absence or presence of TGF-β (1 ng/ml) ± HGF (40 ng/ml) and/or LY294002 (7.5 µM) for 24 hours. Apoptotic index was calculated by counting apoptotic nuclei after PI staining under a fluorescence microscope. A minimum of 1000 nuclei was counted per condition. Data are mean±S.D. of a representative experiment performed with triplicate dishes. **B.** Mouse Met^flx/flx^ and Met^−/−^ oval cells were serum-starved and incubated in the absence or presence of TGF-β (1 ng/ml) ± LY294002 (7.5 µM) for different periods of time. Fluorescence intensity was measured in a FACScan flow cytometer and the percentage of annexin V positive/PI negative cells was calculated. Data are expressed as fold induction over untreated cells and are mean±SEM of four independent experiments. **C, D and E.** Mouse Met^flx/flx^ oval cells were serum-starved and incubated in the absence or presence of TGF-β (1 ng/ml) ± LY294002 (7.5 µM). **C.** After 8 hours of treatment, cells were collected for spectrophotometric determination of intracellular glutathione. Results are expressed as fold change over untreated cells and are mean±SEM of two independent experiments run in duplicate. **D and E.** After 12 hours of treatment RNA was isolated and *Nox4* and *Bmf* mRNA levels were analyzed by qRT-PCR and normalized to the housekeeping gene *Gusb*. Data represent fold change relative to untreated samples and are mean±S.E.M of three independent experiments. **Black bars**, Met^flx/flx^ cells. **White bars**, Met^−/−^ cells. *P<0.05; **P<0.01; ***P<0.001 (treated versus untreated); #*P*<0.05 (T+LY treated versus T treated).

We and others have previously shown that HGF readily activates ERKs MAPK, p38 MAPK, PI3K/AKT, STAT3 and NF-κB in oval cells. Furthermore, some of these pathways have been involved in HGF-mediated proliferative effect on these cells [25], [27], [40], [41], [42]. However, their potential implication in the protective effect of Met revealed in oval cells is not yet known. Certainly, PI3K signaling pathway has long been involved in HGF-mediated cell survival in many cellular contexts including liver cells [25], [43], [44], [45], [46], [47] therefore being a good candidate for analysis. Exogenously added HGF partially suppressed TGF-β-induced apoptosis in Met^flx/flx^ oval cells, effect that was lost when cells were pre-incubated with the PI3K inhibitor LY294002 (figure 5A). These results indicated that PI3K activity is required for HGF anti-apoptotic effect. Furthermore, combined treatment of Met^flx/flx^ oval cells with LY294002 and TGF-β caused an increase in the percentage of apoptotic cells that reached similar levels to those seen in TGF-β-treated Met^−/−^ oval cells, hence, demonstrating that LY294002 sensitizes Met^flx/flx^ oval cells to TGF-β-induced apoptosis mimicking Met^−/−^ oval cells phenotype (figure 5B). Strikingly, the increase in apoptosis observed in Met^flx/flx^ cells treated with LY294002 plus TGF-β was coincident with deeper glutathione depletion (figure 5C) and enhanced TGF-β-mediated up-regulation of *Nox4* and *Bmf* mRNA (figures 5D and 5E). These data provide strong evidence that PI3K impairs TGF-β induced apoptosis by counteracting the oxidative stress induced by this factor.

**Figure 6:**
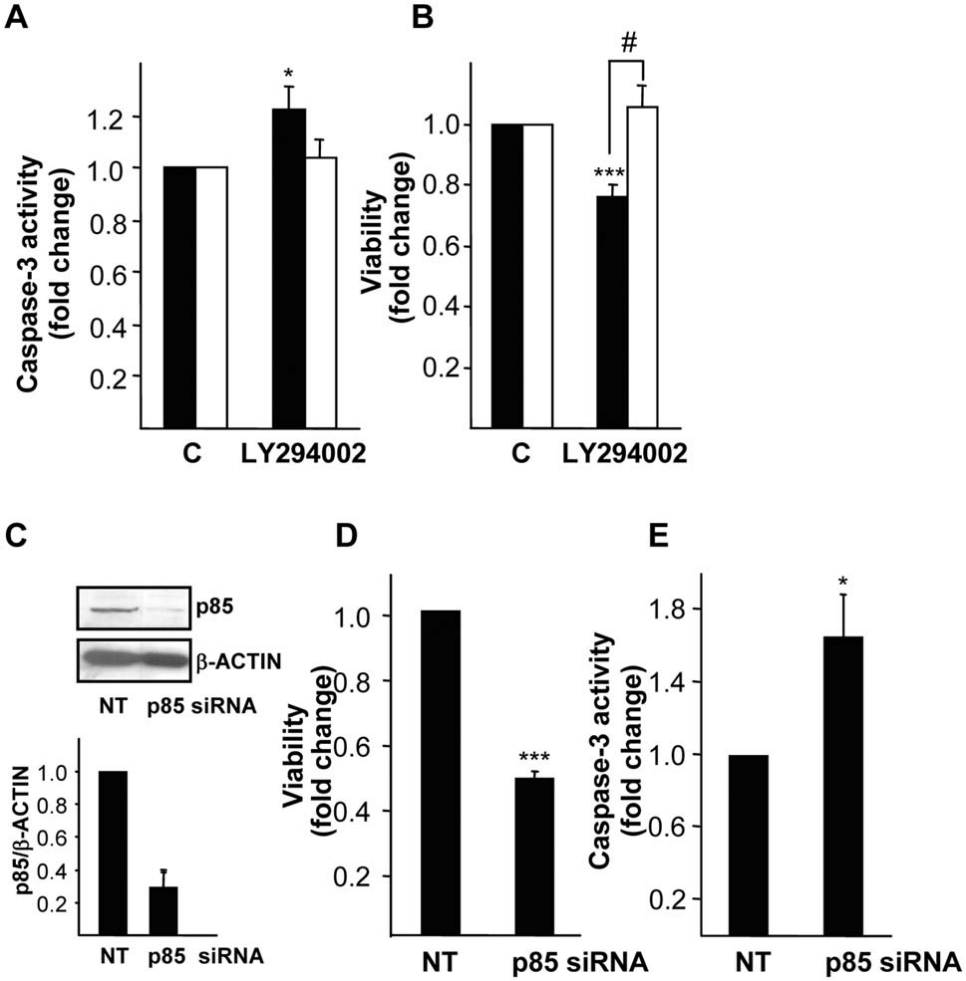
Effect of PI3K inhibition or silencing on basal oval cell viability and apoptosis. A. Mouse Met^flx/flx^ and Met^−/−^ oval cells were serum-starved and incubated in the absence or presence of LY294002 (7.5 µM) for 24 hours. Cell viability was assayed by crystal violet staining. Data are expressed as fold change over untreated cells and are mean±S.D of one representative experiment out of two run in triplicate. **B.** Cells were treated as in B for 15 hours and caspase-3 activity was measured. Data are expressed as fold change over untreated cells and are mean±S.E.M of five independent experiments. **C, D and E.** Mouse Met^flx/flx^ oval cell lines were transfected with non-targeting negative control siRNA (siRNA NT) or p85 targeting siRNA (siRNA p85) for 24 hours and serum-starved for additional 24 hours. **C.** Whole cell lysates were collected and used for immunoblotting with anti-PI3K p85 antibody. β-actin was analyzed as loading control. A representative experiment of five is shown *(upper panel)*. Optical density values relative to loading controls were calculated and expressed as fold change relative to siRNA NT, given an arbitrary value of 1 *(bottom panel*). Data are mean±SEM of five independent experiments. **D.** Caspase-3 activity was analyzed. Data are expressed as fold change over siRNA NT and are mean±SEM of two independent experiments run in duplicate. **E.** Cell viability by crystal violet staining was assayed. Data are expressed as fold change over siRNA NT and are mean±SEM of four independent experiments run in duplicate. **Black bars**, Met^flx/flx^ cells. **White bars**, Met^−/−^ cells. *P<0.05; **P<0.01; ***P<0.001 (treated versus untreated); #*P*<0.05 (treated Met^−/−^ versus treated Met^flx/flx^).

Interestingly, we observed that treatment with LY294002 resulted in an increase in the basal apoptotic index in Met^flx/flx^ oval cell (data not shown). As these experiments were performed in the absence of serum or any exogenously added growth factor, data suggested the existence of a PI3K-mediated autocrine antiapoptotic signaling in oval cells. These data pushed us to explore this effect in more detail. Addition of LY294002 to oval cells had a different effect depending on the presence or the absence of a functional Met receptor. Thus, while PI3K activity inhibition caused a decrease in cell viability and increase in caspase-3 activity in Met^flx/flx^ oval cells, no significant effect was observed in Met^−/−^ oval cells (figures 6A and 6B). The specificity of these effects was subsequently proved by cell transfection with siRNA targeting p85α, the PI3K regulatory subunit. We found that a p85α silencing efficiency of 70% (figure 6C) lead to significant decrease in cell viability and increase in caspase-3 activity (figure 6D and 6E). Together, all these data strongly suggest that PI3K signaling downstream both autocrine and exogenous HGF is responsible for Met-mediated anti-apoptotic activity in oval cells.

## Discussion

In the present study, we have mechanistically addressed the Met-mediated protective effect against TGF-β-triggered apoptosis in mouse oval cells. We have found that autocrine Met-dependent signaling helps counteract TGF-β-induced oxidative stress and mitochondrial dysfunction. Additionally, our results have revealed a pivotal role for PI3K activity in Met-mediated antioxidant and antiapoptotic activities in oval cells.

It has been described that HGF can interfere with SMAD-mediated signaling at different levels. Thus, HGF can negatively regulate SMAD nuclear translocation [48], [49] and SMAD-dependent transcriptional activity by increasing the expression of the SMAD co-repressor SnoN [50]. Therefore, it was plausible that autocrine HGF signaling in Met^flx/flx^ oval cells may impair or modulate TGF-β signaling leading to an impairment of TGF-β-mediated apoptosis. Our results demonstrate that TGF-β readily induces SMAD2 phosphorylation and nuclear translocation in both Met^flx/flx^ and Met^−/−^ oval cells. Although Nguyen *et al* have shown that intensity of SMAD signaling in response to TGF-β in oval cells is weaker than in hepatocytes [15], we prove that the level of induction of the canonical pathway in oval cells is sufficient to control gene transcription. In fact, TGF-β is capable of regulating the expression of prototypical target genes such as plasminogen activator inhibitor-1 (*Pai-1*), inhibitor of differentiation-1 and -2 (*Id1, Id2*) or follistatin (data not shown). Importantly, we could not observe major differences neither in the intensity nor the dynamics of the SMAD signaling between Met^flx/flx^ and Met^−/−^ oval cells, thus discarding a Met-mediated alteration in SMAD signaling in oval cells. Besides SMADs, our results show that JNK and p38 are activated in oval cells in response to TGF-β, providing the first experimental evidence for activation of these signaling pathways by TGF-β in oval cells. However, none of them seems to be required for TGF-β-elicited apoptotic response. These data oppose to previous work in murine hepatocytes (AML12) and rat and human hepatoma cells showing a critical role for these two MAPK in TGF-β-induced apoptosis [51]. Nonetheless, mechanisms mediating TGF-β apoptosis have proven to be cell and context dependent. In line with this, data obtained in mouse oval cells are consistent with previous results from our laboratory using fetal rat hepatocytes, which indicated a ROS-dependent activation of p38 by TGF-β, which is dispensable for apoptosis [52]. Furthermore, epidermal growth factor (EGF) treatment, which blocks TGF-β-induced apoptosis in fetal hepatocytes, does not affect JNK activation by TGF-β, serving as evidence that JNK is not essential in the apoptotic response [53]. Nevertheless, our data cannot rule out a role for p38 and JNK in regulation of other biological effects of TGF-β in oval cells, such as epithelial to mesenchymal transition (EMT) (unpublished results), hypothesis that is being currently tested.

Mechanisms involved in TGF-β-induced apoptosis include the two major apoptotic pathways, death receptor-mediated and mitochondrial apoptosis [28]. Here, we present novel evidence showing that TGF-β-induced apoptosis in oval cells occurs through a mitochondrial-dependent pathway. In our model, TGF-β up-regulates the expression of two pro-apoptotic Bcl-2 family members, Bim and Bmf, as part of the mitochondrial apoptotic program. This is consistent with previous results reported in other cell types [31], [32], [33]. Furthermore, by using gene silencing and other experimental approaches and models, a fundamental role for Bim and Bmf has been demonstrated during TGF-β-induced apoptosis [54], [55], [56]. However, our data show a differential regulation of Bmf in Met^flx/flx^ and Met^−/−^ oval cells, not observed for Bim, which lead us to hypothesize on a differential role for Bmf and Bim during the apoptotic response. In this regard, by using transient knockdown approaches we have demonstrated a critical role for Bmf up-regulation in the oval cell apoptotic response triggered by TGF-β in Met^−/−^ oval cells. Interestingly, our results also show that Bmf, but not Bim up-regulation, is dependent on TGF-β-mediated oxidative stress. This contrasts with previous data reporting that both radical scavengers and Nox4 silencing do impair TGF-β-mediated Bim and Bmf up-regulation, indicating that ROS are involved in the regulation of both genes [31], [33]. Further experiments are needed to clarify the mechanism behind Bim induction by TGF-β in oval cells and its contribution, if any, to the apoptotic response. Pending further investigation, our data provide strong evidence in favor of a relevant role for Bmf in TGF-β induced apoptosis in mouse oval cells.

Our results also strongly suggest that autocrine signaling through Met negatively regulates the increase in Bmf mRNA and protein levels by TGF-β. Little is known about Bmf regulation by growth factors. Up to date, an EGF-dependent Bmf down-regulation has been described in human mammary epithelial cells [57], [58]. No data are currently available regarding potential modulation of Bmf by HGF, although anti-apoptotic signaling through HGF/Met has been largely associated to regulation of members of Bcl-2 family, particularly to induction of anti-apoptotic BCL-2 proteins, including BCL-2 or myeloid cell leukemia-1 (MCL-1) [45], [59]. Awaiting further investigation, we provide novel evidence that negative regulation of pro-apoptotic BCL-2 family proteins, such as BMF, might be an alternative mechanism contributing to Met-dependent anti-apoptotic signaling.

Studies conducted in hepatocytes and HCC cells have demonstrated that oxidative stress is involved in TGF-β-mediated apoptosis in hepatic cells [12], [26], [31], [60], [61]. Additionally, we have proposed a model in which TGF-β-induced ROS have two main sources, mitochondrial and extramitochondrial via Nox4 [31], [36], [38], [39]. Oval cells seem to follow a similar model. Evidence supporting this notion are: i) increased intracellular ROS content and decreased glutathione levels in response to TGF-β; ii) TGF-β-mediated down-regulation of SOD2, catalase and γ-GCS antioxidant enzymes; iii) up-regulation of *Nox4* mRNA preceding the increase in ROS upon TGF-β treatment; iv) impaired activation of caspase-3 and *Bmf* up-regulation in the presence of radical scavengers. More importantly, our data evidence an antioxidant role for HGF/Met axis in oval cells since absence of Met results in an exacerbated oxidative stress process. Thus, Met^−/−^ cells show higher increase in ROS, sustained glutathione depletion and stronger down-regulation of SOD2 and γ-GCS protein and up-regulation of *Nox4* mRNA. All these data undoubtedly show that Met mutant oval cells display a profound redox imbalance in response to TGF-β. This is consistent with previous works reporting that HGF acts as an antioxidant factor able to protect against oxidative stress-induced cell death by increasing the expression and/or activity of γ-GCS and antioxidant enzymes, namely SOD1 and catalase [62], [63], [64]. In addition, similarly to Met^−/−^ oval cells, Met mutant hepatocytes display a dysregulation in oxidative stress-responsive genes and genes involved in glutathione metabolism [65]. Interestingly, HGF not only acts promoting cellular antioxidant defenses but it can also prevent ROS production. Indeed, HGF is able to attenuate the increase in NADPH oxidase activity observed in hippocampus cells after ischemia [66]. Moreover, in mesangial cells exposed to high glucose-mediated oxidative stress HGF exerts a dual antioxidant action, both attenuating the induction of expression of p22(phox), a component of the NADPH oxidase system, and impeding a reduction in γ-GCS expression [67]. Based on our findings, our hypothesis is that something similar might happen in oval cells. As to what are the signaling pathways mediating the pro-survival antioxidant effect of HGF in oval cells, a growing body of evidence suggests that PI3K/AKT pathway is a good candidate. Thus, PI3K/AKT signaling plays a critical role for HGF-mediated protection against CD95- or bile acids-induced apoptosis in primary human and rat hepatocytes, respectively [45], [46]. It also mediates the HGF pro-survival effect on DNA damaging agents-induced apoptosis and oxidative damage caused by ethanol in hepatic tumor cell lines [43], [64]. In oval cells, PI3K signaling pathway has been shown to be required for HGF proliferative effect [41], [42], but nothing is known about its role on the pro-survival effect of HGF. Our results clearly show that treatment with LY294002 completely suppresses the anti-apoptotic effect induced by exogenous HGF demonstrating that PI3K is required for the survival signaling triggered by HGF. Additionally, we show that either LY294002 or siRNA-mediated PI3K silencing by themselves decrease cell viability and increase apoptosis in the absence of serum or any exogenously added growth factor, indicating that PI3K is also mediating autocrine survival signals. We and others have shown that oval cells have an autocrine production of a number of growth factors, including PDGF, EGF and HGF [24], [27], [68], all of them being putative PI3K activators. We have also demonstrated that autocrine signaling through Met and EGFR promotes oval cell survival [24], [27]. The fact that PI3K inhibition or silencing only increased apoptosis in Met^flx/flx^ but not in Met^−/−^ oval cells strongly evidences a Met-dependent PI3K autocrine survival signaling. Besides, PI3K inhibition results in an amplified TGF-β-triggered apoptotic response in Met^flx/flx^ cells resembling that observed in Met^−/−^ cells, with analogous apoptotic indices and importantly, with a magnified oxidative stress and *Nox4* and *Bmf* up-regulation. It has been shown in other cell systems that constitutive activation of AKT by different means, including expression of an active oncogenic mutant catalytic subunit of PI3K (p110α) or active myristoylated AKT1 or AKT3 reduces and/or prevents Bmf induction [57], [69]. Our data strongly suggest that amplification of TGF-β-mediated *Bmf* up-regulation in the presence of LY294002 is a consequence of the enhanced oxidative stress. Additionally, in agreement with previous data in our laboratory reporting that PI3K inhibition increases TGF-β-mediated up-regulation of Nox4 in fetal rat hepatocytes [39], the increased *Nox4* expression in the presence of LY294002 suggest a negative regulatory role for PI3K on Nox4 expression. Although additional work is required to fully characterize the molecular mechanism involved in the regulation of these genes in oval cells, altogether these data allow us to propose a role for PI3K as a critical intracellular negative regulator of Bmf and Nox4 during TGF-β-mediated apoptosis in oval cells.

In summary, both inactivating Met and suppressing PI3K signaling do result in an impairment of oval cell survival. Data presented here constitute the first evidence that the HGF/Met/PI3K signaling axis plays a central role in protecting oval cells against TGF-β-induced oxidative stress and apoptosis. These findings have important implications *in vivo*. Oval cells expand during liver damage and are exposed to apoptotic insults such us TGF-β, which is known to be elevated in several human chronic liver pathologies or other liver injuries involving an oxidative stress process [70]. Hence, antioxidant and anti-apoptotic signaling through Met/PI3K could have a fundamental role in promoting oval cell survival. This mechanism may be relevant for oval cell-mediated liver regeneration but also for its role in HCC development.

## Supporting Information

Figure S1 **Effect of p38 and JNK MAPKs inhibition on TGF-β-induced apoptosis in oval cells.** Mouse Met^flx/flx^ and Met^−/−^ oval cell lines were serum-starved and incubated for 15 hours in the absence or in the presence of 1 ng/ml TGF-β, and in the absence or the presence of (**A**) p38 inhibitor, SB203580 (10 µM) or (**B**) JNK inhibitor SP600125 (30 µM) added 30 minutes before TGF-β. Cells were lysed and caspase-3 activity assayed. Data are mean±SEM of at least three independent experiments.
(PDF)

Figure S2 **Effect of antioxidant agents on intracellular ROS content in oval cells.** Mouse Met^flx/flx^ and Met^−/−^ oval cell lines were serum-starved, pretreated or not with radical scavengers (1 mM ascorbate +50 µM PDTC) for 1 hour prior to TGF-β (1 ng/ml) treatment for 24 hours. After 30 minutes incubation with DFCH-DA (5 µM) fluorescence intensity was measured in a FACScan flow cytometer. Data are expressed as fold induction over untreated cells and are mean±SEM of two independent experiments run in duplicate. **Black bars**, Met^flx/flx^ cells. **White bars**, Met^−/−^ cells. ns = not significant; *P<0.05; **P<0.01 (treated versus untreated); #*P*<0.05 (T treated versus T+Antioxidants treated).
(PDF)

Table S1 **Primers used for qRT-PCR.**
(PDF)
