# Supplementary figures and images for: Mouse Hepatic Oval Cells Require Met-Dependent PI3K to Impair TGF-β-Induced Oxidative Stress and Apoptosis
Source: PLoS One. 2013 Jan 2;8(1):e53108. doi: 10.1371/journal.pone.0053108 (PMC3534654; doi:10.1371/journal.pone.0053108)

**A**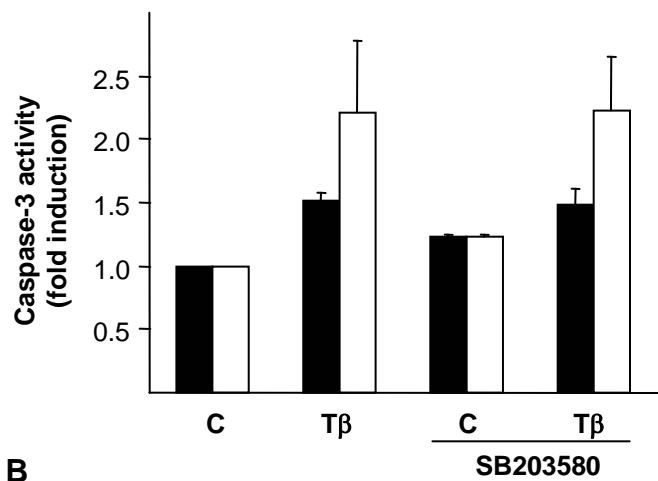**B**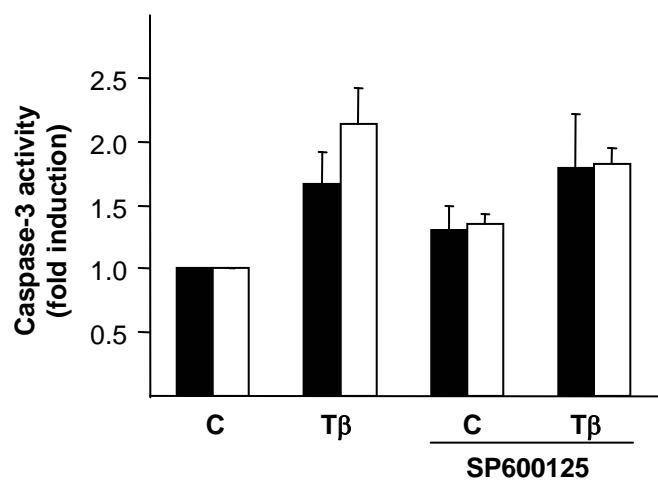

**Supporting Figure 1**

Supplement: Figure S1 — Effect of p38 and JNK MAPKs inhibition on TGF-β-induced apoptosis in oval cells. Mouse Metflx/flx and Met−/− oval cell lines were serum-starved and incubated for 15 hours in the absence or in the presence of 1 ng/ml TGF-β, and in the absence or the presence of (A) p38 inhibitor, SB203580 (10 µM) or (B) JNK inhibitor SP600125 (30 µM) added 30 minutes before TGF-β. Cells were lysed and caspase-3 activity assayed. Data are mean±SEM of at least three independent experiments. (PDF) [file pone.0053108.s001.pdf]

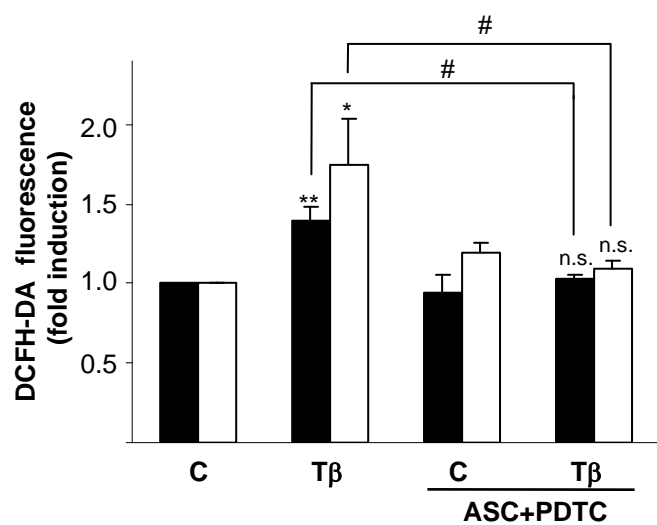

**Supporting Figure 2**

Supplement: Figure S2 — Effect of antioxidant agents on intracellular ROS content in oval cells. Mouse Metflx/flx and Met−/− oval cell lines were serum-starved, pretreated or not with radical scavengers (1 mM ascorbate +50 µM PDTC) for 1 hour prior to TGF-β (1 ng/ml) treatment for 24 hours. After 30 minutes incubation with DFCH-DA (5 µM) fluorescence intensity was measured in a FACScan flow cytometer. Data are expressed as fold induction over untreated cells and are mean±SEM of two independent experiments run in duplicate. Black bars, Metflx/flx cells. White bars, Met−/− cells. ns = not significant; *P<0.05; **P<0.01 (treated versus untreated); #P<0.05 (T treated versus T+Antioxidants treated). (PDF) [file pone.0053108.s002.pdf]
