# Supplementary material for: Mouse Hepatic Oval Cells Require Met-Dependent PI3K to Impair TGF-β-Induced Oxidative Stress and Apoptosis
Source: PLoS One. 2013 Jan 2;8(1):e53108. doi: 10.1371/journal.pone.0053108 (PMC3534654; doi:10.1371/journal.pone.0053108)
Supplement: Table S1 — Primers used for qRT-PCR. (PDF) [file pone.0053108.s003.pdf]

Table S1. Primers used for qRT-PCR

	Forward	*Reverse*
Bim	TGCGCCCGGAGATACGGATT	TGGTCTTCAGCCTCGCGGTA
Bmf	TGTCATGCTGCCTTGTGG	TGAGCCTGCAGGGAAACTG
NOX4	CCTCAACTGCAGCCTCATCC	CAACAATCTTCTTGTTCTCC
Gusb	AAAATGGAGTGCGTGTTGGGT	CCACAGTCCGTCCAGCGCCTT

Supporting Table 1
